# Evolutionary analysis of rabies virus isolates from Guangxi Province of southern China

**DOI:** 10.1186/s12917-018-1514-0

**Published:** 2018-06-18

**Authors:** Xian-Kai Wei, Xiao-Xia He, Yan Pan, Cheng Liu, Hai-Bo Tang, Yi-Zhi Zhong, Xiao-Ning Li, Jing-Jing Liang, Ting Rong Luo

**Affiliations:** 1 State Key Laboratory for Conservation and Utilization of Subtropical Agro-Bioresources, Guangxi University, Nanning, 530004 Guangxi China; 2 Laboratory of Veterinary Microbiology and Animal Infectious Diseases, College of Animal Sciences and Veterinary Medicine, Guangxi University, Nanning, 530004 Guangxi China

**Keywords:** Rabies virus, Genetic diversity, Evolution

## Abstract

**Background:**

Rabies is a severe epidemic in Guangxi province, China, with hundreds of deaths occurring each year. In the past six decades, rabies has emerged three times in Guangxi, and the province has reported the largest number of rabies cases in China. The domestic dog is the principal vector for rabies, and 95% of human cases are associated with transmission from dogs.

**Results:**

To understand the genetic relationship between street rabies virus (RABV) from Guangxi, genetic diversity analysis was performed using RABV isolates collected between 1999 and 2012. The N gene of 42 RABV isolates, and the P and M genes, as well as fragments of the 3′ terminus (L^1–680^) and the polymerase activity module of the L gene (L^pam^) of 36 RABV isolates were sequenced. In addition, whole genome sequencing was performed for 5 RABV isolates. There was evidence of topological discrepancy in the phylogenetic trees based on different genes of the RABV isolates. Amino acid variation of the deduced N protein exhibited different patterns to those obtained from the P and M proteins reported here, and the previously reported G protein (Tang H. et al., PLoS Negl Trop Dis, 8(10): e3114, 2014), and L^1–680^ and L^pam^. These RABV isolates were divided into three main branches against fixed strains.

**Conclusion:**

RABV is prevalent in Guangxi province and strains collected over the last two decades belong mainly to three groups (I, II, III). These RABV isolates reveal genetic diversity. Individual RABV genes from Guangxi exhibit different evolutionary characteristics. The results will have benefits for continuing comprehensive rabies surveillance, prevention and control in China.

**Electronic supplementary material:**

The online version of this article (10.1186/s12917-018-1514-0) contains supplementary material, which is available to authorized users.

## Background

The rabies virus (RABV), a member of the Lyssavirus genus, which is found worldwide, causes lethal viral encephalitis in a wide range of host species. Rabies is of specific concern as it is fatal to humans and has a significant impact on public health.

Previous research on the phylogenetic analysis of the RABV N gene has identified differences in RABVs found in bats, compared to those found in terrestrial mammals [1, 2]. A better understanding of the N gene evolutionary trajectory can assist the development of control measures [3, 4]. Evolutionary analysis based on the lyssavirus surface glycoprotein (ectodomain) revealed that point mutations are the most frequent mutation in lyssavirus evolution; additionally, all lyssavirus lineages have similar evolutionary rates [5]. Phylogenetic analyses of N, P, and G gene sequences among bat lyssaviruses from central Asia have demonstrated that quantitative overlap between the currently established genotypes likely occurs at the amino acid level [6, 7]. Phylogenetic analysis based on the full RABV genome, or based on N, P, M, G, and L genes, resulted in phylogenetic trees with similar topologies, indicating that individual lyssavirus genes are likely sufficient to establish phylogenetic relationships [8]. This suggests that rigorous phylogenetic techniques based on full-length genome sequences provide considerable discriminatory power for genotype classification within the lyssaviruses [9]. The current global understanding of RABV phylogeography is that there are six clades of RABV in non-flying mammals, each with a distinct geographical distribution, likely reflecting major physical barriers to gene flow.

In China, human rabies has re-emerged since 1997. Two distinct clades of RABV isolates found in China were identified by genetic analysis [10, 11]. Investigation of the molecular epidemiology of rabies in southern China has demonstrated that long-distance migration, or trans-provincial movement of dogs (by humans) from high-incidence regions may be one of the causes of the recent human rabies epidemics in southern China [12]. Evolutionary dynamic analysis based on the G gene shows that the RABV currently circulating in China consists of three main groups [13], and that the RABV in China and Southeast Asia share a common ancestor [14]. Phylogenetic analysis based on the 3′ terminus of the N gene shows that RABV isolates from Guangxi province, south China can be divided into four groups [15]. Further studies have demonstrated that isolates from groups I, II, and III are lethal once introduced in mice, whereas isolates of group IV are not fatal to either adult or suckling mice [15].

In this study we sequenced the N, P and M gene as well as L^1–680^ and L^pam^ of the Guangxi isolates. In addition, we performed whole-genome sequencing for 5 of the isolates. The goal of this study was to identify novel genetic features, in several different RABV genes, that may have occurred in the evolutionary process of Guangxi RABV. Due to the fact that live attenuated rabies vaccines have been used in Guangxi in the past, and these vaccines contain RABV strains with the potential for replication and reversion to the pathogenic form, all field isolates were compared against these vaccine strains.

## Methods

### Isolation of virus

Between 1999 and 2012, 42 RABV isolates were obtained from rabid dogs, cattle, pigs, and asymptomatic dogs that were received from different regions of Guangxi. All samples were provided by the Guangxi Center for Animal Diseases Control and Prevention, with permission from the Veterinary Administration of the Guangxi Provincial Government. Samples were subjected to RT-PCR, and the positive samples were further used for RV isolation by mouse inoculation test [15]. Mice were purchased from the Animal Centre of Guangxi Medical University. To comply with Animal Research as Reporting In Vivo Experiments (ARRIVE) guidelines, all husbandry and experimental procedures were conducted in compliance with the Animal Welfare Act and the Guide for the Care and Use of Laboratory Animals. The mice were euthanized in a container after application of halothane inhalant, with the container closed once the aenesthesized mouse displayed a lack of righting reflex (mouse unable to right itself within 10 s after being placed on its side). Reference sequences of Lyssaviruses sequences used for constructing the phylogenetic tree were sourced from GenBank (Additional file 1: Table S1).

### RNA extraction and reverse transcription

Total viral RNA was extracted from original host or mouse brain using Trizol (Invitrogen Biotechnology Co., Ltd, California, America). Following the manufacturer’s instructions, cDNA was synthesized using 2.5 μg total RNA, 1 μL (25 pMol/μL) sense primer of each pair, and 100 U MuMLV RTase (Promega Trading Co., Ltd, Wisconsin, America) in a 25 μL reaction volume using standard methods. Each viral gene or fragment was amplified by RT-PCR using ExTaq (Takala Biomedical Technology Co., Ltd, Dalian, China) DNA polymerase.

### Cloning and sequencing of viral genes

Primers used for amplification of RABV genes are shown in Additional file 2: Table S2. This table includes gene sequences, nucleotide positions, lengths, and regions.

PCR products were separated on 1% agarose and stained with ethidium bromide, purified and cloned into the pMD18-T cloning vector, and sequenced by Takara Corp. Three clones were analyzed for each amplicon of each virus. Sequence information was aligned and edited using DNAStar software.

### Phylogenetic analysis

The coding regions of the N, P, and M genes and the L^1–680^ and L^pam^ regions of the genome of the isolates from Guangxi (accession numbers in Additional file 3: Table S3) were used to construct a phylogenetic tree. Homologous sequences were aligned using the Clustal method of the MegAlign program of DNAStar version 7.1 (DNAS_TAR_ Inc., USA) [16]. A Maximum likelihood (ML) tree for all DNA sequences was constructed using the Kimura 2-parameter model with MEGA5.0 software [17, 18].

## Results

### Phylogenetic analysis of RABV using the N, P, and M genes and regions L^1–680^ and L^pam^

A total of 42 RABV isolates were obtained from different locations in Guangxi between 1999 and 2012. The N of 42 RABV isolates, and the P and M genes, as well as fragments of the 3′ terminus (L^1–680^) and the polymerase activity module of the L gene (L^pam^) of 36 RABV isolates, and the whole genomes of 5 RABV isolates were sequenced (Additional file 3: Table S3). Maximum likelihood phylogenetic trees were constructed for 113 complete N sequences (including the isolates (from Asia) downloaded from GenBank), 84 complete P sequences, 84 complete M sequences, 83 L^1–680^, 83 L^pam^, and 49 whole genome sequences, including a set of laboratory-passaged strains and street RABV strains from other locations (Additional file 1: Table S1).

**Fig. 1:**
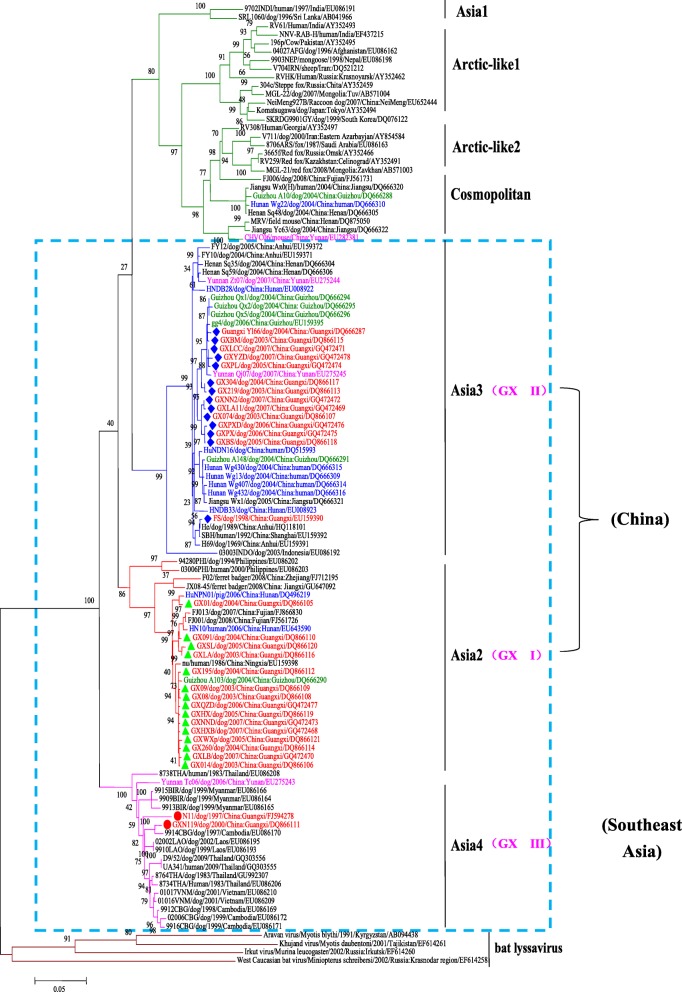
Phylogenetic tree based on nucleotide sequences of N gene

**Fig. 2:**
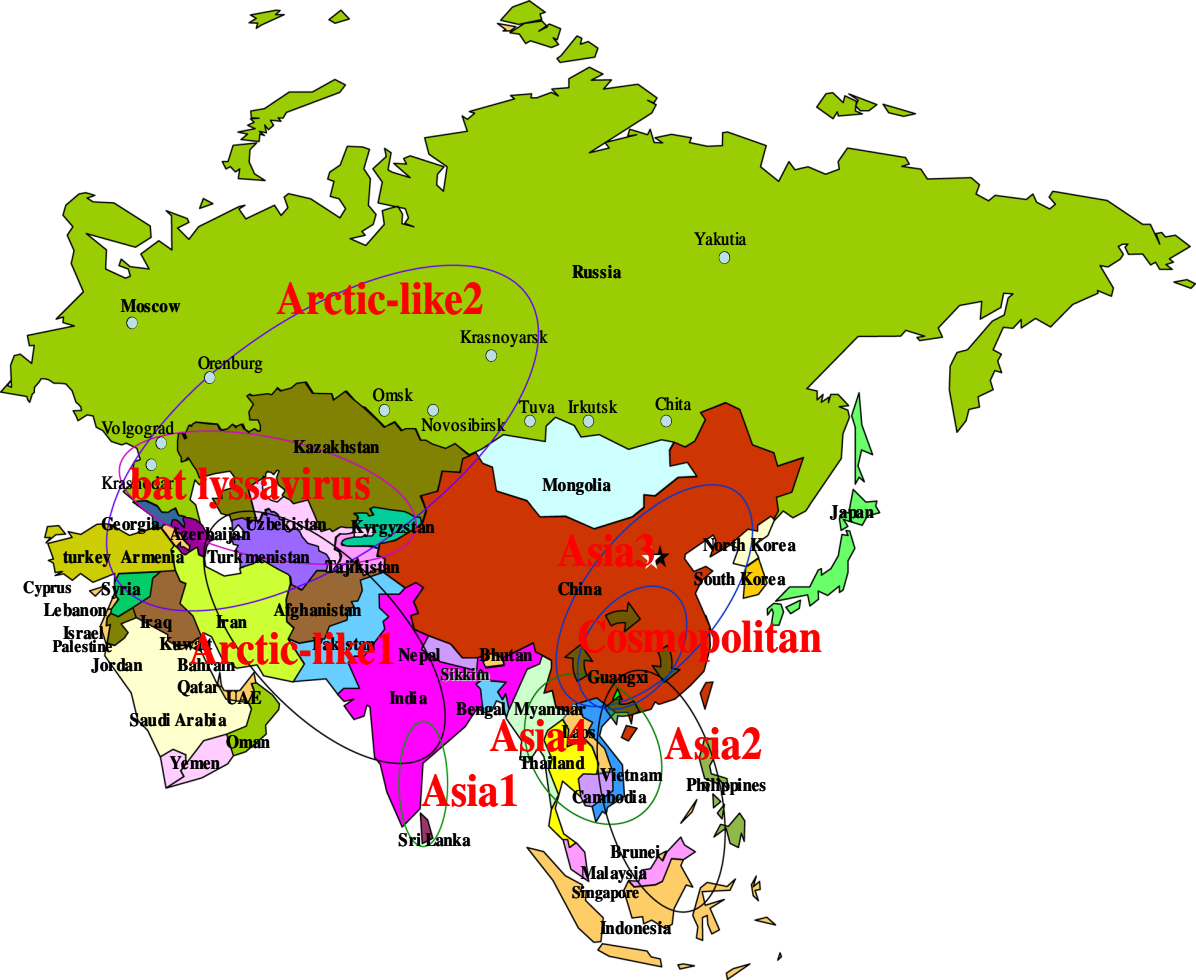
Distribution of RABV isolates from Asia

**Fig. 3:**
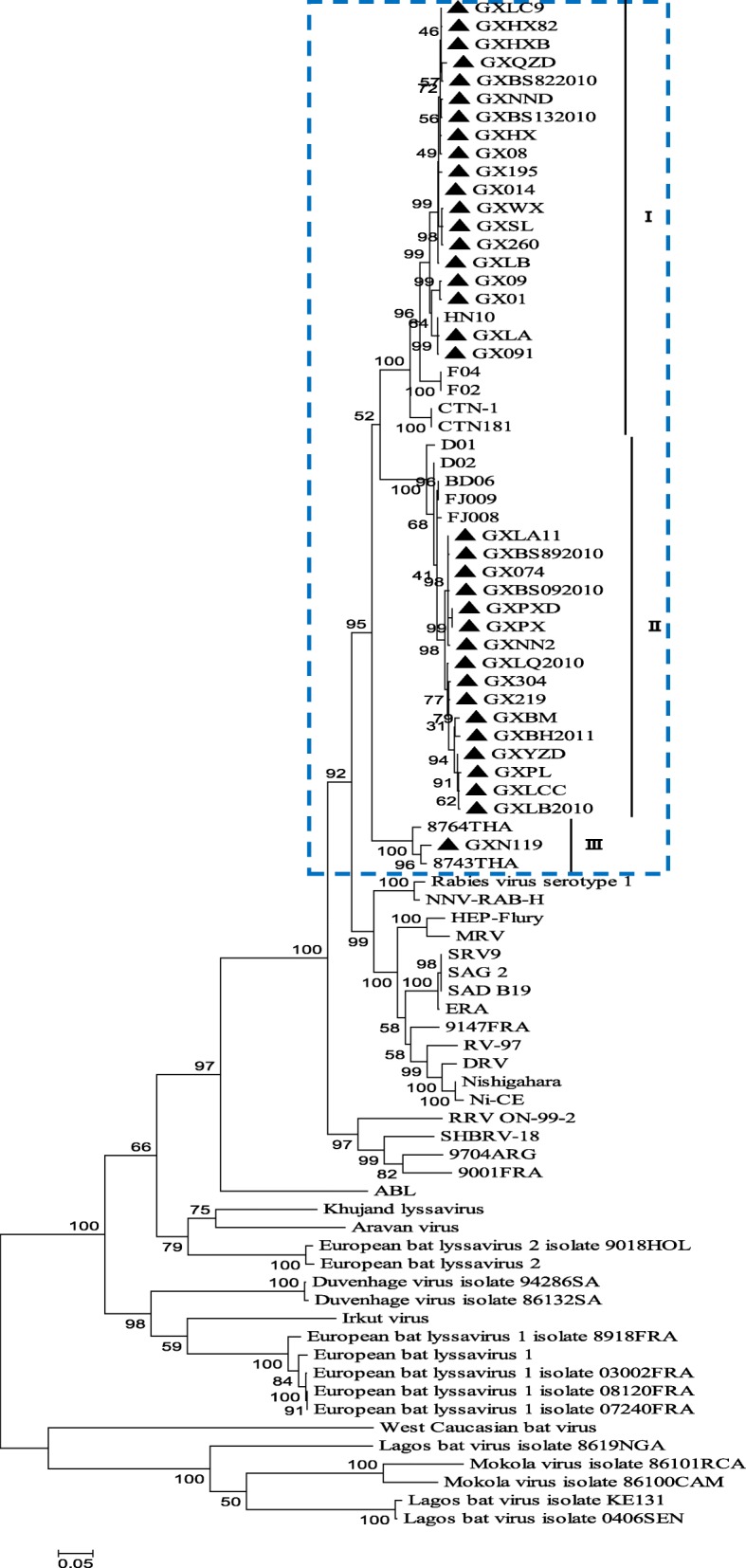
Phylogenetic tree based on nucleotide sequences of the RABV P gene

**Fig. 4:**
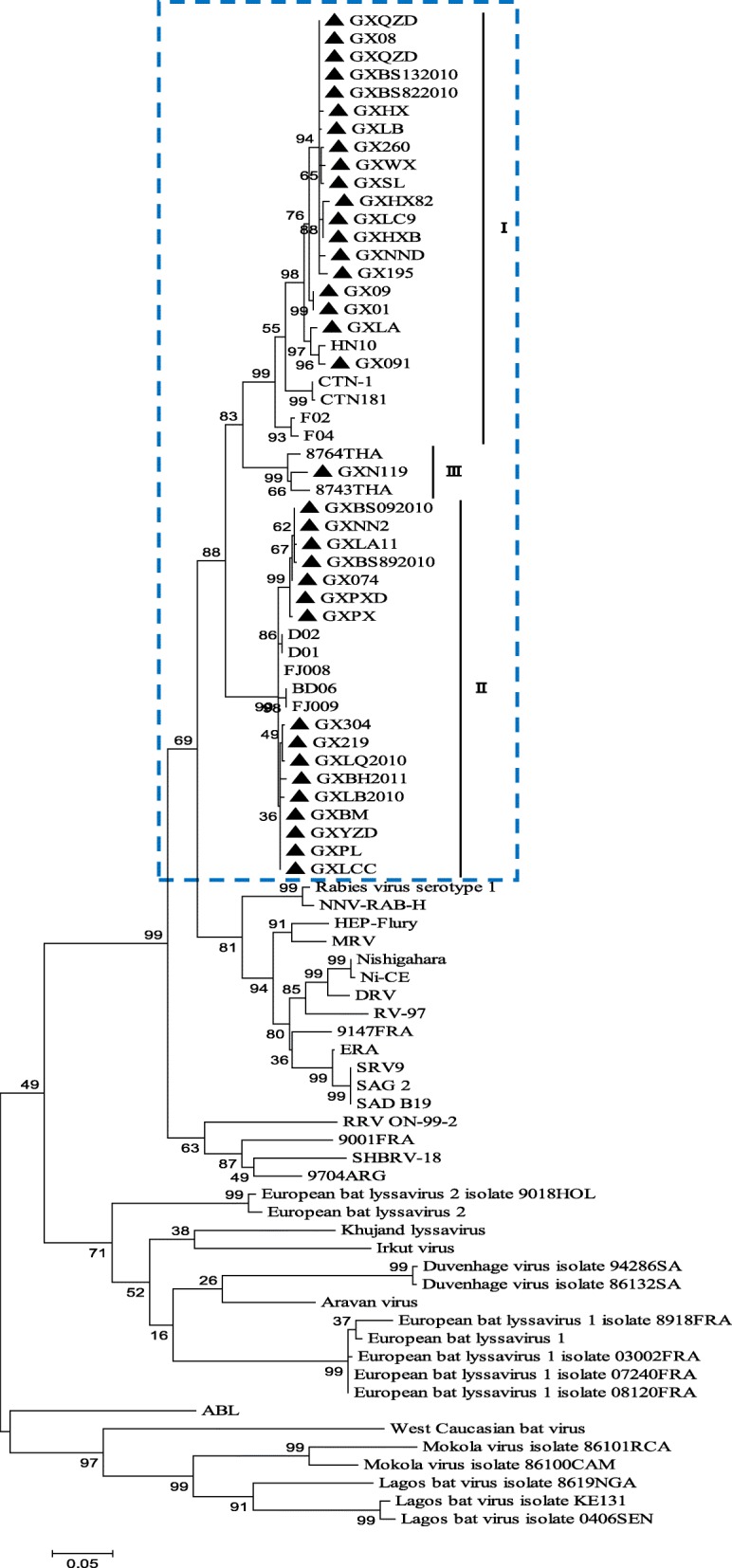
Phylogenetic tree based on nucleotide sequences of the RABV M gene

**Fig. 5:**
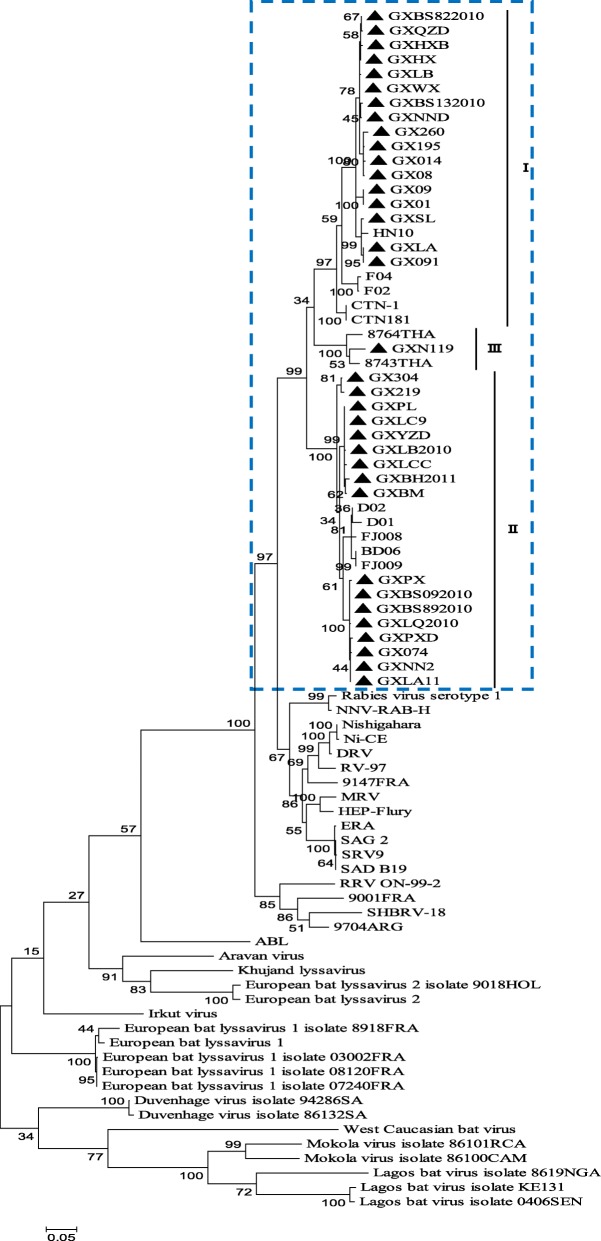
Phylogenetic tree based on nucleotide sequences of the RABV 3′ terminal in the L gene (L^1–680^)

**Fig. 6:**
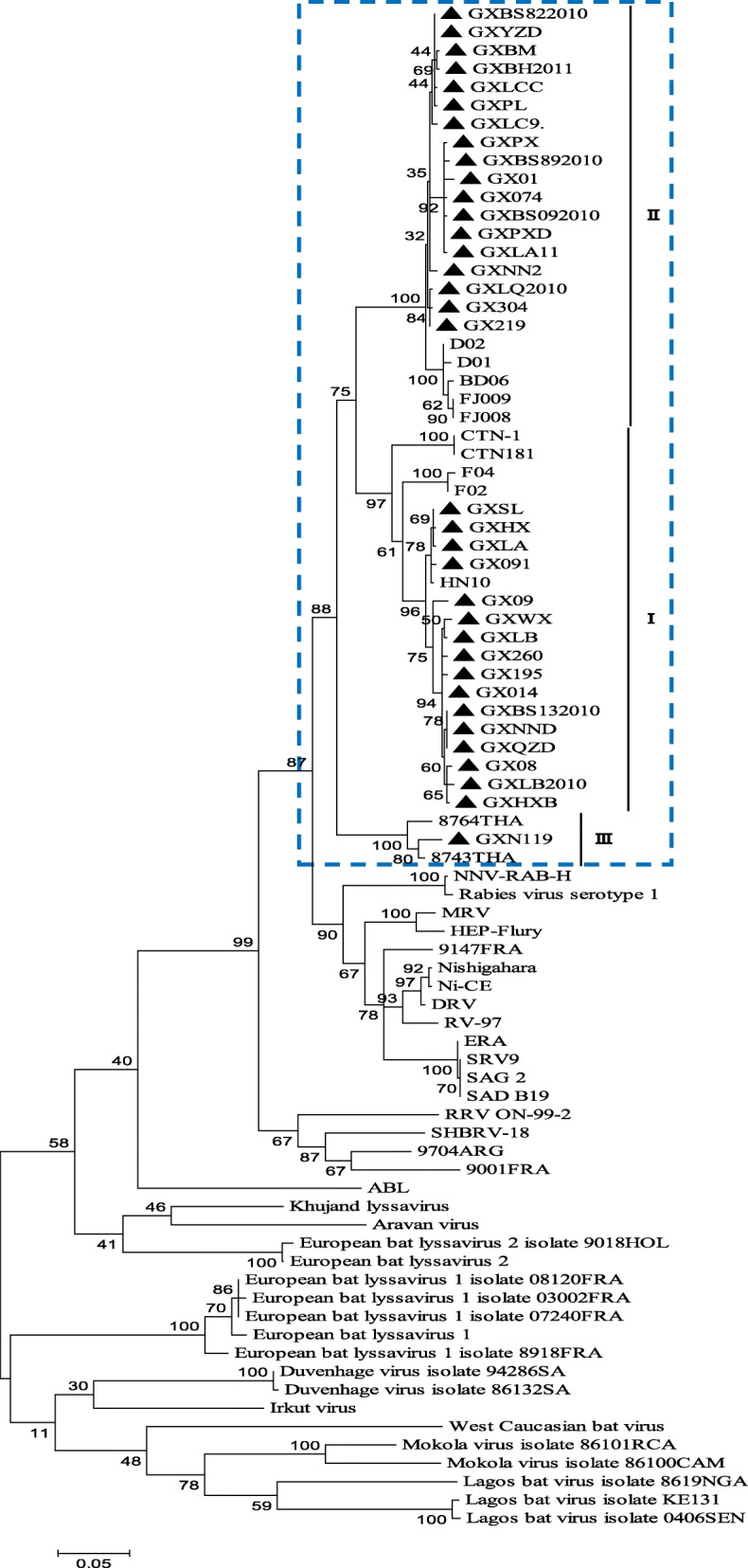
Phylogenetic tree based on nucleotide sequences of the RABV polymerase activity module in the L gene (L^pam^)

**Fig. 7:**
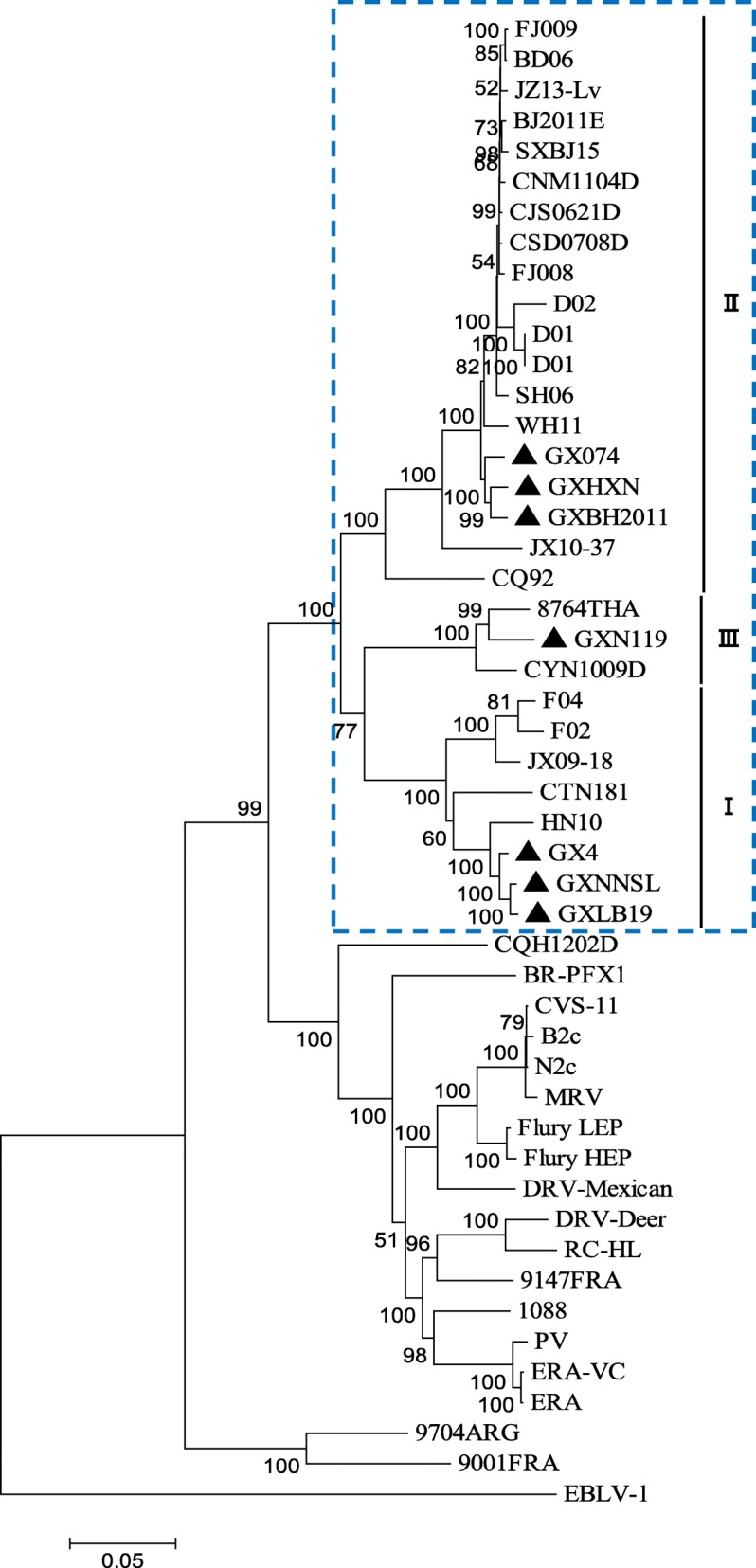
Phylogenetic tree based on RABV whole genome nucleotide sequences

Constructing phylogenetic trees for each of the N, P, and M genes, L^1–680^, L^pam^ and the whole genome, revealed different topologies with strong bootstrap values. Isolates from Guangxi could be divided into three groups, designated I, II, and III (Figs. 1, 3, 4, 5, 6 and 7). Group I included isolates from the Guizhou, Hunan, Jiangxi, Fujian and Zhejiang provinces. Group II contained isolates from the Yunnan, Guizhou, Hunan, Anhui, Jiangsu, and Henan provinces. Group III contained isolates from Guangxi and Yunnan, and from the Southeast Asia countries. Of which, the two main groups (I and II) are related to China I (Clade I) and II (Clade II) [19–21], also associated with Asia1 and Asia2 [14]. We compared the N gene of 113 isolates from the world and constructed the phylogenetic tree, indicating that an India isolate and a Sri Lanka isolate are classified as Asia1, the China I and II are classified as Asia2 and Asia3. Compared with the Asia area, the isolates of group I in Guangxi (GX I) belong to the Asia2, including the isolates from Chinese Hunan, Jiangxi, Zhejiang, Fujian provinces, and including some isolates (94280PHI/dog/1994/Philippines/EU086202 and 03006PHI/human/2000/Philippines/EU086203) from the Philippines. The isolates of group II in Guangxi (GX II) belong to the Asia3, including the isolates from Chinese Yunnan, Guizhou, Hunan, Anhui, Jiangsu, Henan provinces and Shanghai. The RABV isolates of GX I and GX II are principally from the epidemic in Guangxi or from some other provinces of China. The isolates of group III in Guangxi (GX III) belong to Asia4. In the past two decades only three isolates were collected from China, GXN119 was isolated from Guangxi in 2000, and N11 was also from Guangxi, Tc06 from Yunnan; the other isolates were reported in the Southeast Asia countries Thailand, Vietnam, Myanmar, Cambodian and Laos, and were classified as Asia4 [15], indicating that GXIII (Asia4) is mainly prevalent in the Southeast Asia countries (Fig. 2), and the group III is associated with SEA3, as designated in a previous study [21].

Furthermore, the relationship between the three branches varied depending on the gene segment employed. The tree generated from the full N gene, the previously reported G gene [22] and L^pam^ had a branching pattern where groups II and I were linked while group III formed a separate branch. For trees generated from the P gene, groups I and II were clustered most closely while group III formed a separate, outlying branch. However, for the M, L^1–680^ and whole genome trees, groups I and III were most closely related, while group II formed a separate, outlying branch (Figs. 1, 3, 4, 5, 6 and 7 and Table 1).

**Table 1:**
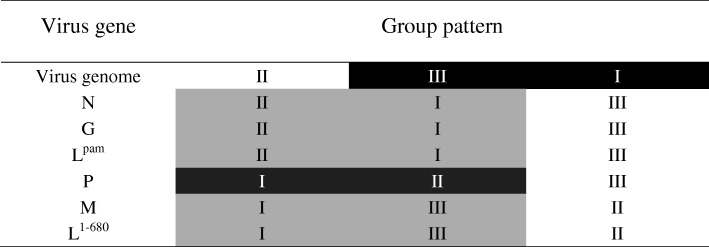
Comparison of genetic patterns based on different genes of rabies virus from Guangxi.

Shading illustrates which groups were most closely related.

### Amino acid variation in RABV isolates

Totally, the nucleotide sequence data from RABV isolates showed that N, M genes and two fragments L^1–680^ and L^pam^ were highly conserved, with the exception that a few nucleotides of the P gene varied. On comparison of deduced amino acid (aa) sequences from N, P, M genes, the fragments of L^1–680^ and L^pam^, as well as the whole genome of 5 isolates, we found that most of the functional motifs mapped in the different genes of RABV isolates were extremely conserved. Details are described as follows.

#### N amino acid variations

Based on the deduced amino acid sequences, the specific epitope (358-367aa) of B cells, the principal epitope (404-418aa) of Th and RNA binding site (298-352aa) were highly conserved [23]. A phosphorylation site S389, related to viral transcription and replication, was also highly conserved [24]. Comparison of the entire N protein of all isolates with that of the ERA, commonly used as a reference strain, showed two specific amino acid substitutions at positions 90 and 110 of group I: T90 N and E110D. In group II, the threonine in ERA was replaced by serine (T42S). The single isolate in group III, GXN119, and isolate 8743THA from Thailand showed 3 different substitutions (L128 V, P135A, and T375 M). In addition, all isolates from Guangxi and other provinces of China had variations at 5 locations that were conserved across regions I, II and III: H26Y, C40S, S61 N, V95 L, and G106D (Additional file 4: Table S4).

#### P amino acid variations

For the P protein, several functional domains -- L protein binding region [25] (1-19aa), LC8 binding motif [26] (144-148aa) and N protein binding region [27] (209-216aa) -- were highly conserved. Nuclear export signal [28] (49-58aa) had two variations: G54R/H57Q in the three groups and one variation E51D in group II. Th/Tc epitope (191-206aa) had one variation, K192E, in the three groups. It is unclear whether the functions of these domains have been influenced or not by these mutations. A phosphorylation site serine at 63aa that was changed to proline (S63P) removed the phosphorylation function.

There were three specific substitutions (A70T, A130M and V131 T) in group I, five specific substitutions (P134S, N135 T, S140P, R151K and A174V) in group II, and six specific substitutions (G73 V, S162 L, Q167K, K239R, D253E, and D281E) in group III. At residue 130, the A130M substitution was specific to group I, while the A130T substitution was specific to group II. At residue 174, the A174V substitution was specific to group II, while the A174E substitution was specific to group III. Four substitutions (H57Q, S63P, S90G, and A241S) were common to all isolates from Guangxi (Additional file 5: Table S5).

Compared with the other genes, P gene also included several sporadic variations in group I and II, resulting in the variation rate of the P gene being higher than the other genes. In group I, four isolates (GXS822010, GXBS13201, GXNNSL and GXLB19) had H57F sporadic variation and three isolates (GXNND, GXLB and GXHX82) had M69 V variation. In group II, there were eight sporadic variations. Of which, five isolates (GXBS89201, GXBA09201, GXLQ2010, GXLB2010 and GXBN2011) had three sporadic variations (H57F, M69G and A70T); four isolates (GXNN2, GXLCC, GXPL, and GXYZD) had four sporadic variations (G61R/K, K62 N, T157I and A170V); four isolates (GX074, GXPX, GXPXD and GXLA11) had one sporadic variation (at N292S); GXHXN isolates had three sporadic variations (H57P, M69G and A70T; Additional file 5: Table S5).

#### M amino acid variations

For the M protein, there were nine substitutions (L26P, S46G, G58E, K77R, S104A, F120 L, I158S, K160R and P175S) common to the three groups I, II and III, one specific substitution I168V in group I, and four specific substitutions (Q17H, S20F, P21S and V22A) in group II. And a functional motif PxSAP [29] mapped at 21-25aa included P21S and V22A variations; its P21S change indicated that the PxSAP motif was abolished in the group II. Another motif PPxY at 35-38aa, relating to viral budding [29], was highly conserved (Additional file 6: Table S6). In addition, the amino acid Y138 that was highly conserved in virulent or avirulent RABV strains was mapped as a functional residue corresponding to viral replication (data not shown).

#### L^1–680^ and the L^pam^ amino acid variations

Alignment of the 226 amino acids of the L^1–680^ fragment revealed four substitutions (R115K, I117M, A168S, and I207V) that were specific to group I, one substitution (T64A) that was specific to group II, and five substitutions (L76F, L88 V, T118A, V157I, and D190E) that were specific to group III. The R48K substitution was common to groups I and II, while V83 L was common to groups II and III. Notably seven substitutions (L2I, L17S, D19A, F73Y, R74K, L136C, and N161 K) were common to all three groups (Table 2).

**Table 2:** Specific amino acid mutations on the 3′ terminal and polymerase activity module of the L gene

Isolate	Group	L^1–680^																				(L^pam^)								
		2	17	19	48	64	73	74	76	83	88	93	115	117	118	136	157	161	168	190	207	49	51	63	72	108	136	145	157	162
ERA		L	L	D	R	T	F	R	L	V	L	M	R	I	T	L	V	N	A	D	I	A	I	R	A	I	Q	R	V	S
GXLA	I	I	S	A	K		Y	K				T	K	M		C		K	S		V	S		W	M	T	R	S	I	A
GX08		I	S	A	K		Y	K				T	K	M		C		K	S		V	S		W	M	T	R	S	I	A
GX09		I	S	A	K		Y	K				T	K	M		C		K	S		V	S		W	M	T	R	S	I	A
GX014		I	S	A	K		Y	K				T	K	M		C		K	S		V	S		W	M	T	R	S	I	A
GX01		I	S	A	K		Y	K				T	K	M		C		K	S		V									
GX091		I	S	A	K		Y	K				T	K	M		C		K	S		V	S		W	M	T	R	S	I	A
GX195		I	S	A	K		Y	K				T	K	M		C		K	S		V	S		W	M	T	R	S	I	A
GX260		I	S	A	K		Y	K				A	K	M		C		K	S		V	S		W	M	T	R	S	I	A
GXHX		I	S	A	K		Y	K				T	K	M		C		K	S		V	S		W	M	T	R	S	I	A
GXWX		I	S	A	K		Y	K				T	K	M		C		K	S		V	S		W	M	T	R	S	I	A
GXSL		I	S	A	K		Y	K				T	K	M		C		K	S		V	S		W	M	T	R	S	I	A
GXQZD		I	S	A	K		Y	K				A	K	M		C		K	S		V	S		W	M	T	R	S	I	A
GXHXB		I	S	A	K		Y	K				T	K	M		C		K	S		V	S		W	M	T	R	S	I	A
GXNND		I	S	A	K		Y	K				A	K	M		C		K	S		V	S		W	M	T	R	S	I	A
GXLB		I	S	A	K		Y	K				A	K	M		C		K	S		V	S		W	M	T	R	S	I	A
GXBS822010		I	S	A	K		Y	K				T	K	M		C		K	S		V	S		W	M	T	R	S	I	A
GXBS132010		I	S	A	K		Y	K				T	K	M		C		K	S		V	S		W	M	T	R	S	I	A
GXYZD	II	I	S	A	K	A	Y	K		L		T				C		K				S	V	W	M	T	R	S	I	
GXLA11		I	S	A	K	A	Y	K		L		T				C		K				S	V	W	M	T	R	S	I	
GXLCC		I	S	A	K	A	Y	K		L		T				C		K				S	V	W	M	T	R	S	I	
GXNN2		I	S	A	K	A	Y	K		L		T				C		K				S	V	W	M	T	R	S	I	
GXPL		I	S	A	K	A	Y	K		L		T				C		K				S	V	W	M	T	R	S	I	
GXPXD		I	S	A	K	A	Y	K		L		T				C		K				S	V	W	M	T	R	S	I	
GX074		I	S	A	K	A	Y	K		L		T				C		K				S	V	W	M	T	R	S	I	
GX219		I	S	A	K	A	Y	K		L		T				C		K				S	V	W	M	T	R	S	I	
GX304		I	S	A	K	A	Y	K		L		T				C		K				S	V	W	M	T	R	S	I	
GXBM		I	S	A	K	A	Y	K		L		T				C		K				S	V	W	M	T	R	S	I	
GXPX		I	S	A	K	A	Y	K		L		T				C		K				S	V	W	M	T	R	S	I	
GXLB2010		I	S	A	K	A	Y	K		L		T				C		K				S	V	W	M	T	R	S	I	
GXBH2011		I	S	A	K	A	Y	K		L		T				C		K				S	V	W	M	T	R	S	I	
GXBS092010		I	S	A	K	A	Y	K		L		T				C		K				S	V	W	M	T	R	S	I	
GXBS892010		I	S	A	K	A	Y	K		L		T				C		K				S	V	W	M	T	R	S	I	
GXLQ2010		I	S	A	K	A	Y	K		L		T				C		K				S	V	W	M	T	R	S	I	
GXN119	III	I	S	A			Y	K	F	L	V	T			A	C	I	K	V	E		S		W	M	T	R	S	I	

Among the 200 amino acids of the L^pam^ fragment we found one substitution, S162A, specific to group I and another, I51V, specific to group II (Table 2).

These data are summarized in Table 2 and Additional file 4: Table S4, Additional file 5: Table S5, Additional file 6: Table S6, which demonstrate that amino acid variations differ across the three groups and serve as viral genetic markers in each of the proteins of wild RABV isolates from Guangxi.

#### Whole genome amino acid variation

The sizes of the individual structural genes, functional motifs and important functional sites of the five RABV isolates for which whole genome sequencing was performed were mostly consistent, with few sites differing. Relative to the coding regions, the non-coding regions were more variable. The full genome of five RABV isolates contained 11,921-11,924 nt in length: 11921 nt/GXLB19; 11,922 nt/GXNNSL and GX4 isolate (from Guangxi downloaded from GenBank); and 11,924 nt/GXBH2011, GXN119, GX074. The P-M non-coding regions were 86-87 nt in length, while the 5’ UTR regions were 129-131 nt in length (Table 3).

**Table 3:** Non-coding regions, coding regions and genome sizes of rabies virus isolates. Numbers represent sizes in base pairs

	GXLB19	GXNNSL	GXBH2011	GXN119	GX074	GX4
3’ UTR	70	70	70	70	70	70
N	1353	1353	1353	1353	1353	1353
N-P	91	91	91	91	91	91
P	894	894	894	894	894	894
P-M	86	86	87	87	87	86
M	609	609	609	609	609	609
M-G	211	211	211	211	211	211
G	1575	1575	1575	1575	1575	1575
G-L	516	516	516	516	516	516
L	6387	6387	6387	6387	6387	6387
5’ UTR	129	130	131	131	131	130
genome	11,921	11,922	11,924	11,924	11,924	11,922

The nAchR-binding region located at 189-214aa of G protein [30] had one common variation E205K in the five isolates. Transcription initiation (AACA) was extremely conserved. In the G-L non-coding region, there are two transcription termination poly:A_7_ (TTP) motifs located downstream of the G protein stop codon. According to the whole genome sequences of the five RABV isolates from Guangxi, the 1st TTP motif (70-77 nt) had 3–5 nt changed, while the second TTP motif located 470-477 nt was conserved and observed in all five isolates (Table 4).

**Table 4:**
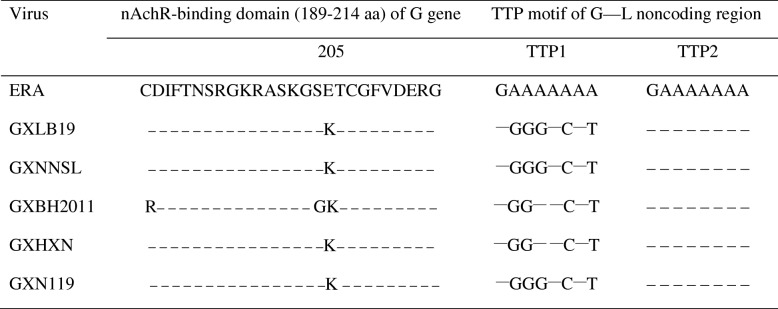
Functional domain of rabies virus

## Discussion

Rabies is a severe epidemic in Guangxi, China, with hundreds of deaths occurring each year [22], and globally the number of rabies cases in China is second only to India [31]. In the past six decades, rabies has emerged three times in Guangxi, which has the largest number of rabies cases in China. The 1st outbreak of rabies was from 1968 to 1976, with the peak of human rabies cases at 560 in 1972. The 2nd time occurred in 1978, reaching a peak of 879 human rabies cases in 1981, and then gradually decreasing to just 24 cases in 1995. However, in the 3rd time, the number of human rabies cases increased from 50 in 1996, rose steeply to 203 in 2003, and reached a peak of 602 in 2004 [22].

The domestic dog is the principal vector for rabies, and 95% of human cases are associated with transmission from dogs. Our research group began surveillance of the RABV-carriage rate of at least 500 clinically healthy dogs from rural areas of Guangxi from 1999, and isolated RABV from RABV-positive cases. Of these isolates, several from rabid and normal dogs taken at different times showed similar pathogenicity in mice, indicating that the RABV from Guangxi has stable virulence [22]. Nevertheless, following an increase in dog vaccination in rural areas, the RABV-carriage rate in dogs appears to be gradually decreasing (data not shown). Between 2013 and 2016, we did not obtain any positive samples even though at least 500 samples from different areas in Guangxi were tested each year. Therefore, the data shown in this study consists of RABV isolates collected up until 2012. After reviewing a molecular epidemiological study conducted in Guangxi [15], we decided to further examine the genetic properties of these RABV isolates.

Generally, similar phylogenetic trees imply similar evolutionary paths [32–34]. In the phylogenetic analysis of Wu et al., trees constructed with the N, P, M, G, and L genes were found to have similar topologies [8]. In this study, the phylogenetic trees constructed from the complete N, P, and M genes as well as the L^1–680^ and L^pam^ fragments exhibited different topologies with strong bootstrap values as well as G gene (Table 1) [22], although the Guangxi RABV isolates of three groups were closely related (Figs. 1, 3, 4, 5, 6, and 7). The possible reason might be a difference of variation rate (varied nucleotides/total nucleotides of each gene) in each gene [35, 36]. The comprehensive datasets of the N, P, M, L^1–680^, and L^pam^ genes, widely distributed across different provinces of China, corroborated the assumption that the Guangxi RABV genes were likely undergoing co-evolution.

It is worth mentioning that although phylogenetic trees of the N, P, and M genes and the L^1–680^ and L^pam^ fragments consistently had three groups, branching of the three viral groups actually revealed four different patterns. The tree generated from the N, G gene and L^pam^ has a branching pattern in which groups II (Asia 3) and I (Asia2) are linked with group III (Asia4) lying on a separate branch; the trees generated from P gene had groups I and II clustered most closely with group III forming an outlying branch; trees generated from the M and L^1–680^ genes and whole genome sequences had groups I and III most closely related, with group II as an outlying branch. This finding indicates that the rate of evolution might vary depending on the RABV gene examined (Table 1).

Based on the phylogenetic analysis and amino acid variation, the RABV isolates from Guangxi were divided into two main groups (I and II) and a minor group (III). However, the phylogenetic trees constructed from the complete N, P and M genes as well as the L^1–680^ and L^pam^ fragments exhibited different topologies with strong bootstrap values as well as G gene. In fact, most of the isolates from the south China including Hunan, Guizhou, Guangdong, Fujian, Jiangsu, Anhui and Henan provinces belong to two main groups, the same as those isolates from Guangxi. Nevertheless, some isolates from Hunan, Guizhou, Jiangsu, Anhui, and Henan provinces are classified into the group Cosmopolitan (Fig. 1).

On the basis of differences identified by the phylogenetic analysis of nucleotide sequences, the downstream significance of these variations at the protein level was explored. Protein alignment revealed several specific amino acid residue mutations in each protein, which coincided with the phylogenetic analysis and grouping of isolates from Guangxi. Construction of phylogenetic trees and comparison of amino acids of each protein revealed an overall picture of significant genetic diversity for the isolates from Guangxi.

In this study, we present the sequences of the entire N, P, and M genes and the L^1–680^ and L^pam^ fragments of the L gene from 42 RABV isolates from Guangxi. We previously also sequenced the full length G gene for which we performed phylogenetic analysis [22]. The final results revealed that Maximum likelihood (ML) of the N gene sequences generated a tree with a similar overall structure to that obtained with the G gene and L^pam^ sequences [22], while different to that obtained using P, M gene and L^1–680^. In addition, the deduced amino acid sequences of the N, P, and M proteins and L^1–680^ and L^pam^ for all 42 isolates from Guangxi were aligned and compared to other terrestrial strains. Variations in the N protein demonstrated different patterns to those obtained for the P and M proteins, and for the L^1–680^ and L^pam^ fragments of the L protein, as well as for the G protein [22]. RNA variation in RABV isolates from Guangxi revealed a significant genetic diversity with differences observed from other geographical regions, and there are strong geographical associations among RABV isolates including the three groups in Guangxi.

Although live attenuated oral rabies vaccines could revert to virulent strains and have residual pathogenicity in certain rodents, they have been shown to be safe in foxes, dogs and skunks [37, 38]. Intensive molecular investigation of SAD B19 strain passaged several times in mice has demonstrated their sequence conservation and genetic stability in vivo [39]. However, there is always a risk that vaccine strains could revert to a fully pathogenic form, and thus, all RABV isolates collected from the field should be sequenced and subjected to phylogenetic analysis. We found that the attenuated vaccine ERA strain used across Guangxi was genetically distributed in a different group compared with the Guangxi RABV isolates (Figs. 1, 3, 4, 5, 6 and 7).

Here, we performed a detailed genetic analysis of RABV isolates from Guangxi. These data provide rich genetic information on nucleotide and amino acid variation patterns in RABV proteins and reveal genetic diversity in the RABV epidemic in Guangxi.

## Additional files

Additional file 1: **Table S1.** Reference sequences of Lyssaviruses used in the present study. (DOC 282 kb)

Additional file 2: **Table S2.** Primers used in this study. (DOC 184 kb)

Additional file 3: **Table S3.** Origin of rabies virus isolates from Guangxi used in this study. (DOC 288 kb)

Additional file 4: **Table S4.** Specific amino acids mutations in the nucleoprotein (N) of rabies virus isolates from Guangxi. (DOCX 19 kb)

Additional file 5: **Table S5.** Specific mutational amino acid on phosphoprotein (P) protein of rabies virus isolates from Guangxi. (DOCX 23 kb)

Additional file 6: **Table S6.** Specific mutational amino acid on matrix protein of rabies virus isolates from Guangxi. (DOCX 22 kb)

## Abbreviations

G: Glycoprotein
L^1–680^: 3′ terminal 1–680 amino aicd in L gene
L^pam^: Polymerase activity module in L gene
M: Matrix protein
N: Nucleoprotein
P: Phosphoprotein
RABV: Rabies virus
