# Supplementary material for: Evolutionary analysis of rabies virus isolates from Guangxi Province of southern China
Source: BMC Vet Res. 2018 Jun 18;14:188. doi: 10.1186/s12917-018-1514-0 (PMC6006964; doi:10.1186/s12917-018-1514-0)
Supplement: Supplementary file 1 — Table S1. Reference sequences of Lyssaviruses used in the present study. (DOC 282 kb) [file 12917_2018_1514_MOESM1_ESM.doc]

Supplemental Table 1 Reference sequences of Lyssaviruses used in the present study

| Isolate | District | Host | GenBank accession number | | | | |  |
| --- | --- | --- | --- | --- | --- | --- | --- | --- |
|  |  |  | N gene | P gene | M gene | 3’ terminal of  L gene | Polymerase activity module of L gene | Whole genome |
| BD06 | China: Hebei | Dog | EU549783 | EU549783 | EU549783 | EU549783 | EU549783 | EU549783 |
| FJ009 | China: Fujian | Dog | FJ866836 | FJ866836 | FJ866836 | FJ866836 | FJ866836 | FJ866836 |
| FJ008 | China: Fujian | Dog | FJ866835 | FJ866835 | FJ866835 | FJ866835 | FJ866835 | FJ866835 |
| D01 | China: Zhejiang | Dog | FJ712193 | FJ712193 | FJ712193 | FJ712193 | FJ712193 | FJ712193 |
| D02 | China: Zhejiang | Dog | FJ712194 | FJ712194 | FJ712194 | FJ712194 | FJ712194 | FJ712194 |
| HN10 | China: Hunan |  | EU643590 | EU643590 | EU643590 | EU643590 | EU643590 | EU643590 |
| CTN181 | China: Shandong | Vaccine strain | EF564174 | EF564174 | EF564174 | EF564174 | EF564174 | EF564174 |
| CTN-1 | China: Shandong | Vaccine strain | FJ959397 | FJ959397 | FJ959397 | FJ959397 | FJ959397 | FJ959397 |
| F04 | China: Zhejiang | Chinese ferret badger | FJ712196 | FJ712196 | FJ712196 | FJ712196 | FJ712196 | FJ712196 |
| F02 | China: Zhejiang | Chinese ferret badger | FJ712195 | FJ712195 | FJ712195 | FJ712195 | FJ712195 | FJ712195 |
| SXBJ15 | China: Shaanxi | Cattle | KR230089 | KR230089 | KR230089 | KR230089 | KR230089 | KR230089 |
| BJ2011E | China: Beijing | Equine | JQ423952 | JQ423952 | JQ423952 | JQ423952 | JQ423952 | JQ423952 |
| CNM1104D | China: Inner mongllia | Dog | KC252634 | KC252634 | KC252634 | KC252634 | KC252634 | KC252634 |
| JZ13-Lv | China: Hebei | Human | KJ004416 | KJ004416 | KJ004416 | KJ004416 | KJ004416 | KJ004416 |
| CJS0261D | China: Jiangsu | Dog | JQ970481 | JQ970481 | JQ970481 | JQ970481 | JQ970481 | JQ970481 |
| CSD0708D | China: Shandong | Dog | JQ970486 | JQ970486 | JQ970486 | JQ970486 | JQ970486 | JQ970486 |
| SH06 | China: Shanghai | Dog | GU345748 | GU345748 | GU345748 | GU345748 | GU345748 | GU345748 |
| WH11 | China: Hubei | Donkey | JQ647510 | JQ647510 | JQ647510 | JQ647510 | JQ647510 | JQ647510 |
| JX10-37 | China: Jiangxi | Ferret badger | KF726953 | KF726953 | KF726953 | KF726953 | KF726953 | KF726953 |
| CQ92 | China: Chongqing | Dog | GU345746 | GU345746 | GU345746 | GU345746 | GU345746 | GU345746 |
| JX09-18 | China: Jiangxi | Ferret badger | KF726852 | KF726852 | KF726852 | KF726852 | KF726852 | KF726852 |
| CYN1009D | China: Yunnan | Dog | JQ730682 | JQ730682 | JQ730682 | JQ730682 | JQ730682 | JQ730682 |
| MRV | China: Henan | Mouse | DQ875050 | DQ875050 | DQ875050 | DQ875050 | DQ875050 | DQ875050 |
| CQH1202D | China: Qinghai | Dog | KM272192 | KM272192 | KM272192 | KM272192 | KM272192 | KM272192 |
| 1088 | USA: Atlanta | Woodchuck | AB645847 | AB645847 | AB645847 | AB645847 | AB645847 | AB645847 |
| 8743THA | Thailand | Human | EU293121 | EU293121 | EU293121 | EU293121 | EU293121 | EU293121 |
| 8764THA | Thailand | Homo sapiens | EU293111 | EU293111 | EU293111 | EU293111 | EU293111 | EU293111 |
| Ni-CE | Japan | Lab strain | AB128149 | AB128149 | AB128149 | AB128149 | AB128149 | AB128149 |
| Nishigahara | Japan | Lab strain | AB044824 | AB044824 | AB044824 | AB044824 | AB044824 | AB044824 |
| RV-97 | Russia | Vaccine strain | EF542830 | EF542830 | EF542830 | EF542830 | EF542830 | EF542830 |
| DRV | China: Jilin | Deer | DQ875051 | DQ875051 | DQ875051 | DQ875051 | DQ875051 | DQ875051 |
| 9147FRA | France | Fox | EU293115 | EU293115 | EU293115 | EU293115 | EU293115 | EU293115 |
| SAD B19 | USA | Vaccine strain | M31046 | M31046 | M31046 | M31046 | M31046 | M31046 |
| SAG 2 | France | Vaccine strain | EF206719 | EF206719 | EF206719 | EF206719 | EF206719 | EF206719 |
| SRV9 | China | Vaccine strain | AF499686 | AF499686 | AF499686 | AF499686 | AF499686 | AF499686 |
| ERA | USA | Vaccine/dog | EF206707 | EF206707 | EF206707 | EF206707 | EF206707 | EF206707 |
| ERA-VC | China | Vaccine strain | FJ913470 | FJ913470 | FJ913470 | FJ913470 | FJ913470 | FJ913470 |
| HEP-Flury | Japan | Vaccine strain | AB085828 | AB085828 | AB085828 | AB085828 | AB085828 | AB085828 |
| MRV | China: Henan | Mouse | DQ875050 | DQ875050 | DQ875050 | DQ875050 | DQ875050 | DQ875050 |
| NNV-RAB-H | India | Homo sapiens | EF437215 | EF437215 | EF437215 | EF437215 | EF437215 | EF437215 |
| Rabies virus serotype 1 | Germany |  | AY956319 | AY956319 | AY956319 | AY956319 | AY956319 | AY956319 |
| 9704ARG | Argentina | Tadarida brasiliensis | EU293116 | EU293116 | EU293116 | EU293116 | EU293116 | EU293116 |
| SHBRV-18 | USA | Silver-haired bat | AY705373 | AY705373 | AY705373 | AY705373 | AY705373 | AY705373 |
| 9001FRA | Guyana | Dog | EU293113 | EU293113 | EU293113 | EU293113 | EU293113 | EU293113 |
| RRV ON-99-2 | Canada | Procyon lotor | EU311738 | EU311738 | EU311738 | EU311738 | EU311738 | EU311738 |
| Abl | Australia | Bat | AF418014 | AF418014 | AF418014 | AF418014 | AF418014 | AF418014 |
| European bat lyssavirus 1 isolate 07240FRA | France | Eptesicus serotinus | EU626552 | EU626552 | EU626552 |  |  |  |
| European bat lyssavirus 1 isolate 08120FRA | France | Eptesicus serotinus | EU626551 | EU626551 | EU626551 |  |  |  |
| European bat lyssavirus 1 isolate 03002FRA | France | Eptesicus serotinus | EU293109 | EU293109 | EU293109 | EU293109 | EU293109 | EU293109 |
| European bat lyssavirus 1 | Germany | Eptesicus serotinus | NC_009527 | NC_009527 | NC_009527 | NC_009527 | NC_009527 | NC_009527 |
| European bat lyssavirus 1 isolate 8918FRA | France | Eptesicus serotinus | EU293112 | EU293112 | EU293112 | EU293112 | EU293112 | EU293112 |
| Duvenhage virus isolate 86132SA | South Africa | Homo sapiens | EU293119 | EU293119 | EU293119 | EU293119 | EU293119 | EU293119 |
| Duvenhage virus isolate 94286SA | South Africa | Miniopterus | EU293120 | EU293120 | EU293120 | EU293120 | EU293120 | EU293120 |
| Irkut virus | Russia | Murina leucogaster | EF614260 | EF614260 | EF614260 | EF614260 | EF614260 | EF614260 |
| European bat lyssavirus 2 isolate 9018HOL | Netherlands | Myotis dasycneme | EU293114 | EU293114 | EU293114 | EU293114 | EU293114 | EU293114 |
| European bat lyssavirus 2 | United Kingdom | Homo sapiens | NC_009528 | NC_009528 | NC_009528 | NC_009528 | NC_009528 | NC_009528 |
| Khujand lyssavirus | Tajikistan |  | EF614261 | EF614261 | EF614261 | EF614261 | EF614261 | EF614261 |
| Aravan virus | Kyrgyzstan |  | EF614259 | EF614259 | EF614259 | EF614259 | EF614259 | EF614259 |
| Lagos bat virus isolate 0406SEN | Senegal | Eidolon helvum | EU293108 | EU293108 | EU293108 | EU293108 | EU293108 | EU293108 |
| Lagos bat virus isolate KE131 | Kenya | Eidolon helvum | EU259198 | EU259198 | EU259198 | EU259198 | EU259198 | EU259198 |
| Lagos bat virus isolate 8619NGA | Nigeria | Eidolon helvum | EU293110 | EU293110 | EU293110 |  |  |  |
| Mokola virus isolate 86100CAM | Cameroon | Shrew | EU293117 | EU293117 | EU293117 | EU293117 | EU293117 | EU293117 |
| Mokola virus isolate 86101RCA | Central African Republic | Rodent | EU293118 | EU293118 | EU293118 | EU293118 | EU293118 | EU293118 |
| West Caucasian bat virus | Russia | Miniopterus schreibersi | EF614258 | EF614258 | EF614258 | EF614258 | EF614258 | EF614258 |
