# Supplementary material for: Evolutionary analysis of rabies virus isolates from Guangxi Province of southern China
Source: BMC Vet Res. 2018 Jun 18;14:188. doi: 10.1186/s12917-018-1514-0 (PMC6006964; doi:10.1186/s12917-018-1514-0)
Supplement: Supplementary file 2 — Table S2. Primers used in this study. (DOC 184 kb) [file 12917_2018_1514_MOESM2_ESM.doc]

Supplemental Table 2 Primers used in this study

| Primers | Sense | Sequences (5'-3') | Nucleotide position | Length  (bp) | Region for gene |
| --- | --- | --- | --- | --- | --- |
| RV1-F | + | ACGCTTAACAACMARAYCAA | 1-20 | 495 | 3' terminus |
| RV1-R | — | CCGACTAAAGATGCATGCTC | 476-495 |  |  |
| RV2-F | + | ACAGACAGCGTCAATTGCAAAGC | 28-50 | 1512 | N gene |
| RV2-R | — | TCGGATTGACGAAGATCTTGCTC | 1517-1539 |  |  |
| RV3-F | + | GACTTAGCACTGGCAGACGAC | 1202-1222 | 353 | N-P intergenic region |
| RV3-R | — | CGGCTCTGATTGCACT | 1539-1554 |  |  |
| RV4-F | + | CACCCCTCCTTTCGAACCATC | 1488-1508 | 1072 | P gene |
| RV4-R | — | GCGGAGAAAGGCTTATGAGTG | 2540-2559 |  |  |
| RV5-F1 | + | TTGAAGATGAACCTCGACGAC | 2202-2222 | 1259 | M gene |
| RV5-R1 | — | ACAACCAAATTATTCGGACAG | 3440-3460 |  |  |
| RV6-F | + | ATCCCTCAAAAGACTCAAGG | 3293-3312 | 1818 | G gene |
| RV6-R | — | CCGTTAGTCACTGAAACTGC | 5091-5110 |  |  |
| RV7-F | + | GGTCATATCTTCATGGGAATCATA | 4838-4861 | 609 | G-L intergenic region |
| RV7-R | — | TCAACTGGGTCATCATAAACCTCT | 5423-5446 |  |  |
| RV8-F | + | GAACAACTGGCAACACTTCTCAAC | 5368-5391 | 905 | L1 gene |
| RV8-R | — | GGACAAAACTTGATCCCCAGCTAT | 6249-6272 |  |  |
| RV9-F | + | TTTATCTCGTTTCAACTCTCT | 6158-6178 | 1092 | L2 gene |
| RV9-R | — | GCATATGTAACCCTTGAATAG | 7229-7249 |  |  |
| RV10-F | + | CTACATCTTGCCACTTTTTGACGC | 7130-7153 | 1167 | L3 gene |
| RV10-R | — | CTAGAAGCCTAGTGAAGCTCTC | 8272-8296 |  |  |
| RV11-F | + | TGGATTCATGCCCTGTGTCAGG | 8214-8235 | 1309 | L4 gene |
| RV11-R | — | TTAGAGATTCAAAATCTCCGGG | 9501-9522 |  |  |
| RV12-F1 | + | TTCTCAGATCTTCGAGTTTCC | 9401-9421 | 1402 | L5 gene |
| RV12-R1 | — | TGCATCACAAATGATGAGGTC | 10782-10802 |  |  |
| RV13-F | + | TGGAAGTACTTCCAGTCAGTCC | 10737-10758 | 1130 | L6 gene |
| RV13-R | — | GATTCACGATCTTGTTTTTTTC | 11845-11866 |  |  |
| RV14-F | + | CTCAAAAGCAGGTCATTCGAGGG | 11521-11543 | 402 | 5' terminus |
| RV14-R | — | GCTCCGACCCACGCTTAACAAA | 11911-1 |  |  |
