# Supplementary material for: Evolutionary analysis of rabies virus isolates from Guangxi Province of southern China
Source: BMC Vet Res. 2018 Jun 18;14:188. doi: 10.1186/s12917-018-1514-0 (PMC6006964; doi:10.1186/s12917-018-1514-0)
Supplement: Supplementary file 3 — Table S3. Origin of rabies virus isolates from Guangxi used in this study. (DOC 288 kb) [file 12917_2018_1514_MOESM3_ESM.doc]

Supplemental Table 3 Origin of rabies virus isolates from Guangxi used in this study

| Isolate | District | Host | Collection date | GenBank accession number | | | | |  |
| --- | --- | --- | --- | --- | --- | --- | --- | --- | --- |
|  |  |  |  | N gene | P gene | M gene | 3’ terminal of  L gene | Polymerase activity module of L gene | Whole genome |
| GXN119 | Nanning | Dog | 2000.10 | DQ 866111 | GQ 472497 | GQ 472525 | GQ 472578 | GQ 472604 | MG201920 |
| GXLA | Nanning | Dog | 2003.01 | DQ 866116 | GQ 472494 | GQ 472522 | GQ 472575 | GQ 472601 | — |
| GX074 | Baise | Dog | 2003.02 | DQ 866107 | GQ 472483 | GQ 472511 | GQ 472565 | GQ 472591 | MG201923 |
| GX08 | Qinzhou | Dog | 2003.03 | DQ 866108 | GQ 472480 | GQ 472508 | GQ 472562 | GQ 472588 | — |
| GX09 | Qinzhou | Dog | 2003.03 | DQ 866109 | GQ 472481 | GQ 472509 | GQ 472563 | GQ 472589 | — |
| GXBM | Hechi | Dog | 2003.03 | DQ 866115 | GQ 472489 | GQ 472517 | GQ 472571 | GQ 472597 | — |
| GX014 | Chongzuo | Dog | 2003.04 | DQ 866106 | GQ 472482 | GQ 472510 | GQ 472564 | GQ 472590 | — |
| GX219 | Guigang | Dog | 2003.06 | DQ 866113 | GQ 472486 | GQ 472514 | GQ 472568 | GQ 472594 | — |
| GX304 | Fangchenggang | Dog | 2004.03 | DQ 866117 | GQ 472488 | GQ 472516 | GQ 472570 | GQ 472596 | — |
| GX01 | Guilin | Dog | 2004.03 | DQ 866105 | GQ 472479 | GQ 472507 | GQ 472561 | — | — |
| GX091 | Liuzhou | Dog | 2004.07 | DQ 866110 | GQ 472484 | GQ 472512 | GQ 472566 | GQ 472592 | — |
| GX195 | Wuzhou | Dog | 2004.10 | DQ 866112 | GQ 472485 | GQ 472513 | GQ 472567 | GQ 472593 | — |
| GX260 | Laibin | Dog | 2004.12 | DQ 866114 | GQ 472487 | GQ 472515 | GQ 472569 | GQ 472595 | — |
| GXHX | Hengxian | Dog | 2005.03 | DQ 866119 | GQ 472491 | GQ 472519 | GQ 472572 | GQ 472598 | — |
| GXSL | Shanglin | Cattle | 2005.03 | DQ 866120 | GQ 472504 | GQ 472532 | GQ 472585 | GQ 472611 | — |
| GXPX | Pinxiang | Dog | 2005.03 | GQ 472475 | GQ 472501 | GQ 472529 | GQ 472582 | GQ 472608 | — |
| GXWX | Wuxuan | Pig | 2005.03 | DQ 866121 | GQ 472505 | GQ 472533 | GQ 472586 | GQ 472612 | — |
| GXBS | Baise | Dog | 2005.04 | DQ 866118 | — | — | — | — | — |
| GXPXD | Pinxiang | Dog | 2006.08 | GQ 472476 | GQ 472502 | GQ 472530 | GQ 472583 | GQ 472609 | — |
| GXQZD | Qinzhou | Dog | 2006.08 | GQ 472477 | GQ 472503 | GQ 472531 | GQ 472584 | GQ 472610 | — |
| GXHXB | Hengxian | Dog | 2007.04 | GQ 472468 | GQ 472492 | GQ 472520 | GQ 472573 | GQ 472599 | — |
| GXLCC | Liucheng | Dog | 2007.04 | GQ 472471 | GQ 472496 | GQ 472524 | GQ 472577 | GQ 472603 | — |
| GXPL | Panlong | Dog | 2007.04 | GQ 472474 | GQ 472500 | GQ 472528 | GQ 472581 | GQ 472607 | — |
| GXYZD | Yizhou | Dog | 2007.04 | GQ 472478 | GQ 472506 | GQ 472534 | GQ 472587 | GQ 472613 | — |
| GXNND | Nanning | Dog | 2007.04 | GQ 472473 | GQ 472499 | GQ 472527 | GQ 472580 | GQ 472606 | — |
| GXNN2 | Nanning | Dog | 2007.08 | GQ 472472 | GQ 472498 | GQ 472526 | GQ 472579 | GQ 472605 | — |
| GXLB | Liubei | Dog | 2007.08 | GQ 472470 | GQ 472495 | GQ 472523 | GQ 472576 | GQ 472602 | — |
| GXLA11 | Longan | Dog | 2007.11 | GQ 472469 | GQ 472493 | GQ 472521 | GQ 472574 | GQ 472600 | — |
| GXHX82 | Hengxian | Dog | 2007.11 | — | GQ 472490 | GQ 472518 | — | — | — |
| GX4 | Guangxi | Dog | 1994 | GU 358653 | GU 358653 | GU 358653 | GU 358653 | GU 358653 | GU 358653 |
| GXLQ2010 | Liangqing | Dog | 2010.05 | MG201876 | MG201888 | MG201892 | MG201912 | MG201915 | — |
| GXLB2010 | Liubei | Dog | 2010.09 | MG201877 | MG201885 | MG201893 | MG201908 | MG201916 | — |
| GXBS092010 | Tianyang | Dog | 2010.11 | MG201881 | MG201890 | MG201891 | MG201907 | MG201918 | — |
| GXBS132010 | Tianyang | Dog | 2010.11 | MG201880 | MG201887 | MG201896 | MG201910 | MG201914 | — |
| GXBS822010 | Xilin | Dog | 2010.11 | MG201879 | MG201886 | MG201895 | MG201911 | MG201913 | — |
| GXBS892010 | Xilin | Dog | 2010.11 | MG201878 | MG201889 | MG201894 | MG201909 | MG201917 | — |
| GXBH2011 | Haicheng | Dog | 2011.05 | MG201922 | MG201922 | MG201922 | MG201922 | MG201922 | MG201922 |
| GXNNSL | Shanglin | Dog | 2011.12 | MG201919 | MG201919 | MG201919 | MG201919 | MG201919 | MG201919 |
| GXLZ04 | Luzhai | Dog | 2011.12 | MG201882 | — | — | — | — | — |
| GXRA03 | Rongan | Dog | 2012.02 | MG201875 | — | — | — | — | — |
| GXRA06 | Rongan | Dog | 2012.02 | MG201884 | — | — | — | — | — |
| GXHCHJ | Rongan | Dog | 2012.03 | MG201883 | — | — | — | — | — |
| GXLB19 | Xingbin | Dog | 2012.03 | MG201921 | MG201921 | MG201921 | MG201921 | MG201921 | MG201921 |

Notes: L^1-680^, 1-680 nucleotides at 3’ terminal of L gene; L^pam^, polymerase activity module of L gene.
