# Supplementary material for: Evolutionary analysis of rabies virus isolates from Guangxi Province of southern China
Source: BMC Vet Res. 2018 Jun 18;14:188. doi: 10.1186/s12917-018-1514-0 (PMC6006964; doi:10.1186/s12917-018-1514-0)
Supplement: Supplementary file 4 — Table S4. Specific amino acids mutations in the nucleoprotein (N) of rabies virus isolates from Guangxi. (DOCX 19 kb) [file 12917_2018_1514_MOESM4_ESM.docx]

Supplemental Table 4 Specific amino acids mutations in the nucleoprotein (N) of rabies virus isolates from Guangxi

| Strains | Group | Position of amino acid mutations | | | | | | | | | | | |
| --- | --- | --- | --- | --- | --- | --- | --- | --- | --- | --- | --- | --- | --- |
|  |  | 26 | 40 | 42 | 61 | 90 | 95 | 106 | 110 | 128 | 135 | 375 | 379 |
| **ERA** |  | **H** | **C** | **T** | **S** | **T** | **V** | **G** | **E** | **L** | **P** | **T** | **V** |
| GXLA | I | Y | S |  | N | N | L | D | D |  | S |  | L |
| GX08 |  | Y | S |  | N | N | L | D | D |  | S |  | L |
| GX09 |  | Y | S |  | N | N | L | D | D |  | S |  | L |
| GX014 |  | Y | S |  | N | N | L | D | D |  | S |  | L |
| GX01 |  | Y | S |  | N | N | L | D | D |  | S |  | L |
| GX091 |  | Y | S |  | N | N | L | D | D |  | S |  | L |
| GX195 |  | Y | S |  | N | N | L | D | D |  | S |  | L |
| GX260 |  | Y | S |  | N | N | L | D | D |  | S |  | L |
| GXHX |  | Y | S | A | N | N | L | D | D |  | S |  | L |
| GXWX |  | Y | S |  | N | N | L | D | D |  | S |  | L |
| GXSL |  | Y | S |  | N | N | L | D | D |  | S |  | L |
| GXQZD |  | Y | S |  | N | N | L | D | D |  | S |  | L |
| GXHXB |  | Y | S |  | N | N | L | D | D |  | S |  | L |
| GXNND |  | Y | S |  | N | N | L | D | D |  | S |  | L |
| GXLB |  | Y | S |  | N | N | L | D | D |  | S |  | L |
| GXHX82 |  | Y | S |  | N | N | L | D | D |  | S |  | L |
| GXBS132010 |  | Y | S |  | N | N | L | D | D |  | S |  | L |
| GXLB19 |  | Y | S |  | N | N | L | D | D |  | S |  | L |
| GXNNSL |  | Y | S |  | N | N | L | D | D |  | S |  | L |
| GXRA06 |  | Y | S |  | N | N | L | D | D |  | S |  | L |
| GXBS82201 |  | Y | S |  | N | N | L | D | D |  | S |  | L |
| GX074 | II | Y | S | S | N |  | L | D |  |  | S |  | L |
| GXBM |  | Y | S | S | N |  | L | D |  |  | S |  | L |
| GX219 |  | Y | S | S | N |  | L | D |  |  | S |  | L |
| GX304 |  | Y | S | S | N |  | L | D |  |  | S |  | L |
| GXPX |  | Y | S | S | N |  | L | D |  |  | S |  | L |
| GXPXD |  | Y | S | S | N |  | L | D |  |  | S |  | L |
| GXLCC |  | Y | S | S | N |  | L | D |  |  | S |  | L |
| GXPL |  | Y | S | S | N |  | L | D |  |  | S |  | L |
| GXYZD |  | Y | S | S | N |  | L | D |  |  | S |  | L |
| GXNN2 |  | Y | S | S | N |  | L | D |  |  | S |  | L |
| GXLA11 |  | Y | S | S | N |  | L | D |  |  | S |  | L |
| GXLB2010 |  | Y | S | S | N |  | L | D |  |  | S |  | L |
| GXBH2011 |  | Y | S | S | N |  | L | D |  |  | S |  | L |
| GXLQ2010 |  | Y | S | S | N |  | L | D |  |  | S |  | L |
| GXBS092010 |  | Y | S | S | N |  | L | D |  |  | S |  | L |
| GXBS892010 |  | Y | S | S | N |  | L | D |  |  | S |  | L |
| GXLZ04 |  | Y | S | S | N |  | L | D |  |  | S |  | L |
| GXRA03 |  | Y | S | S | N |  | L | D |  |  | S |  | L |
| GXHCHJ |  | Y | S | S | N |  | L | D |  |  |  |  | L |
| GXN119 | III | Y | S |  | N |  | L | D | E | V | A | M |  |
| 8743THA |  | Y | S |  | N |  | L | D | E | V | A | M |  |
