# Supplementary material for: Evolutionary analysis of rabies virus isolates from Guangxi Province of southern China
Source: BMC Vet Res. 2018 Jun 18;14:188. doi: 10.1186/s12917-018-1514-0 (PMC6006964; doi:10.1186/s12917-018-1514-0)
Supplement: Supplementary file 5 — Table S5. Specific mutational amino acid on phosphoprotein (P) protein of rabies virus isolates from Guangxi. (DOCX 23 kb) [file 12917_2018_1514_MOESM5_ESM.docx]

Supplemental Table 5 Specific mutational amino acid on phosphoprotein (P) protein of rabies virus isolates from Guangxi

| Strain | Group | **Amino acid** **position** | | | | | | | | | | | | | | | | | | | | | | | | |
| --- | --- | --- | --- | --- | --- | --- | --- | --- | --- | --- | --- | --- | --- | --- | --- | --- | --- | --- | --- | --- | --- | --- | --- | --- | --- | --- |
|  |  | 57 | 61 | 62 | 63 | 64 | 69 | 70 | 73 | 90 | 130 | 131 | 134 | 135 | 140 | 151 | 157 | 162 | 167 | 170 | 174 | 239 | 241 | 253 | 281 | 292 |
| **ERA** |  | **H** | **G** | **K** | **S** |  | **M** | **A** | **G** | **S** | **A** | **V** | **P** | **N** | **S** | **R** | **T** | **S** | **Q** | **A** | **A** | **K** | **A** | **D** | **D** | **N** |
| GXLA | Ⅰ | Q |  |  | P |  | V | T |  | G | M | T |  |  |  |  |  |  |  |  |  |  | S |  |  |  |
| GX08 |  | Q |  |  | P |  | V | T |  | G | M | T |  |  |  |  |  |  |  |  |  |  | S |  |  |  |
| GX09 |  | Q |  |  | P |  | V | T |  | G | M | T |  |  |  |  |  |  |  |  |  |  | S |  |  |  |
| GX014 |  | Q |  |  | P |  | V | T |  | G | M | T |  |  |  |  |  |  |  |  |  |  | S |  |  |  |
| GX01 |  | Q |  |  | P |  | V | T |  | G | M | T |  |  |  |  |  |  |  |  |  |  | S |  |  |  |
| GX091 |  | Q |  |  | P |  | V | T |  | G | M | T |  |  |  |  |  |  |  |  |  |  | S |  |  |  |
| GX195 |  | Q |  |  | P |  | V | T |  | G | M | T |  |  |  |  |  |  |  |  |  |  | S |  |  |  |
| GX260 |  | Q |  |  | P |  | V | T |  | G | M | T |  |  |  |  |  |  |  |  |  |  | S |  |  |  |
| GXHX |  | Q |  |  | P |  | V | T |  | G | M | T |  |  |  |  |  |  |  |  |  |  | S |  |  |  |
| GXWX |  | Q |  |  | P |  | V | T |  | G | M | T |  |  |  |  |  |  |  |  |  |  | S |  |  |  |
| GXSL |  | Q |  |  | P |  | V | T |  | G | M | T |  |  |  |  |  |  |  |  |  |  | S |  |  |  |
| GXQZD |  | Q |  |  | P |  | V | T |  | G | M | T |  |  |  |  |  |  |  |  |  |  | S |  |  |  |
| GXHXB |  | Q |  |  | P |  | V | T |  | G | M | T |  |  |  |  |  |  |  |  |  |  | S |  |  |  |
| GXNND |  | Q |  |  | P |  | A | T |  | G | M | T |  |  |  |  |  |  |  |  |  |  | S |  |  |  |
| GXLB |  | Q |  |  | P |  | A | T |  | G | M | T |  |  |  |  |  |  |  |  |  |  | S |  |  |  |
| GXHX82 |  | Q |  |  | P |  | A | T |  | G | M | T |  |  |  |  |  |  |  |  |  |  | S |  |  |  |
| GXS822010 |  | F |  |  | P |  | V | T |  | G | M | T |  |  |  |  |  |  |  |  |  |  | S |  |  |  |
| GXBS132010 |  | F |  |  | P |  | V | T |  | G | M | T |  |  |  |  |  |  |  |  |  |  | S |  |  |  |
| GXNNSL |  | F |  |  | P |  | V | T |  | G | M | T |  |  |  |  |  |  |  |  |  |  | S |  |  |  |
| GXLB19 |  | F |  |  | P |  | V | T |  | G | M | T |  |  |  |  |  |  |  |  |  |  | S |  |  |  |
| GX074 | Ⅱ | Q |  |  | P |  |  |  |  | G | T |  | S | T | P | K |  |  |  |  | V |  | S |  |  | S |
| GXPX |  | Q |  |  | P |  |  |  |  | G | T |  | S | T | P | K |  |  |  |  | V |  | S |  |  | S |
| GXPXD |  | Q |  |  | P |  |  |  |  | G | T |  | S | T | P | K |  |  |  |  | V |  | S |  |  | S |
| GXLA11 |  | Q |  |  | P |  |  |  |  | G | T |  | S | T | P | K |  |  |  |  | V |  | S |  |  | S |
| GXNN2 |  | Q | R | N | P |  |  |  |  | G | T |  | S | T | P | K | I |  |  | V | V |  | S |  |  |  |
| GXLCC |  | Q | R | N | P |  |  |  |  | G | T |  | S | T | P | K | I |  |  | V | V |  | S |  |  |  |
| GXPL |  | Q | K | N | P |  |  |  |  | G | T |  | S | T | P | K | I |  |  | V | V |  | S |  |  |  |
| GXYZD |  | Q | R | N | P |  |  |  |  | G | T |  | S | T | P | K | I |  |  | V | V |  | S |  |  |  |
| GX219 |  | Q |  |  | P |  |  |  |  | G | T |  | S | T | P | K |  |  |  |  | V |  | S |  |  |  |
| GXBM |  | Q |  |  | P |  |  |  |  | G | T |  | S | T | P | K |  |  |  |  | V |  | S |  |  |  |
| GX304 |  | Q |  |  | P |  |  |  |  | G | T |  | S | T | P | K |  |  |  |  | V |  | S |  |  |  |
| GXBS892010 |  | F |  |  | P |  | G | T |  |  |  |  | S | T | P | K |  |  |  |  | V |  | S |  |  |  |
| GXBS092010 |  | F |  |  | P |  | G | T |  |  |  |  | S | T | P | K |  |  |  |  | V |  | S |  |  |  |
| GXLQ2010 |  | F |  |  | P |  | G | T |  |  |  |  | S | T | P | K |  |  |  |  | V |  | S |  |  |  |
| GXLB2010 |  | F |  |  | P |  | G | T |  |  |  |  | S | T | P | K |  |  |  |  | V |  | S |  |  |  |
| GXBH2011 |  | F |  |  | P |  | G | T |  |  |  |  | S | T | P | K | S | L |  |  | V |  | S |  |  |  |
| GXHXN |  | P |  |  |  |  | G | T |  |  |  |  |  |  |  |  |  |  |  |  |  |  |  |  |  |  |
| GXN119 | Ⅲ | Q |  |  | P |  | I |  | V |  |  |  |  |  |  |  |  | L | K |  | E | R | S | E | E |  |
