# Supplementary material for: Evolutionary analysis of rabies virus isolates from Guangxi Province of southern China
Source: BMC Vet Res. 2018 Jun 18;14:188. doi: 10.1186/s12917-018-1514-0 (PMC6006964; doi:10.1186/s12917-018-1514-0)
Supplement: Supplementary file 6 — Table S6. Specific mutational amino acid on matrix protein of rabies virus isolates from Guangxi. (DOCX 22 kb) [file 12917_2018_1514_MOESM6_ESM.docx]

Supplemental Table 6 Specific mutational amino acid on matrix protein of rabies virus isolates from Guangxi

| **Strain** | **Group** | **Amino acid position** | | | | | | | | | | | | | | | | | | | |
| --- | --- | --- | --- | --- | --- | --- | --- | --- | --- | --- | --- | --- | --- | --- | --- | --- | --- | --- | --- | --- | --- |
|  |  |  |  |  | **PxSAP motif** | | | | |  |  |  |  |  |  |  | ***** |  |  |  |  |
|  |  | **13** | 17 | 20 | 21 | 22 | 23 | 24 | 25 |  | **26** | 46 | 58 | **77** | 104 | 120 | **138** | **158** | **160** | 168 | **175** |
| **ERA** |  | **D** | **Q** | **S** | **P**  **p** | **V** | **S** | **A** | **P** |  | **L** | **S** | **G** | **K** | **S** | **F** | **Y** | **I** | **K** | **I** | **P** |
| GXLA | Ⅰ |  |  |  |  |  |  |  |  |  | P | G | E | R | A | L |  | S | R | V | S |
| GX08 |  |  |  |  |  |  |  |  |  |  | P | G | E | R | A | L |  | S | R | V | S |
| GX09 |  |  |  |  |  |  |  |  |  |  | P | G | E | R | A | L |  | S | R | V | S |
| GX014 |  |  |  |  |  |  |  |  |  |  | P | G | E | R | A | L |  | S | R | V | S |
| GX01 |  |  |  |  |  |  |  |  |  |  | P | G | E | R | A | L |  | S | R | V | S |
| GX091 |  |  |  |  |  | A |  |  |  |  | P | G | E | R | A | L |  | S | R | V | S |
| GX195 |  |  |  |  | S |  |  |  |  |  | P | G | E | R | A | L |  | S | R | V | S |
| GX260 |  |  |  |  |  |  |  |  |  |  | P | G | E | R | A | L |  | S | R | V | S |
| GXHX |  |  |  |  |  |  |  |  |  |  | P | G | E | R | A | L |  | S | R | V | S |
| GXWX |  |  |  |  |  |  |  |  |  |  | P | G | E | R | A | L |  | S | R | V | S |
| GXSL |  |  |  |  |  |  |  |  |  |  | P | G | E | R | A | L |  | S | R | V | S |
| GXQZD |  |  |  |  |  |  |  |  |  |  | P | G | E | R | A | L |  | S | R | V | S |
| GXHXB |  |  |  |  |  |  |  |  |  |  | P | G | E | R | A | L |  | S | R | V | S |
| GXNND |  |  |  |  |  |  |  |  |  |  | P | G | E | R | A | L |  | S | R | V | S |
| GXLB |  |  |  |  |  |  |  |  |  |  | P | G | E | R | A | L |  | S | R | V | S |
| GXHX82 |  |  |  |  |  |  |  |  |  |  | P | G | E | R | A | L |  | S | R | V | S |
| GXS822010 |  |  |  |  |  |  |  |  |  |  | P | G | E | R | A | L |  | S | R | V | S |
| GXBS132010 |  |  |  |  |  |  |  |  |  |  | P | G | E | R | A | L |  | S | R | V | S |
| GX074 | Ⅱ |  | H | F | S | A |  |  |  |  | P | G | E | R | A | L |  | S | R |  | S |
| GXBM |  |  | H | F | S | A |  |  |  |  | P | G | E | R | A | L |  | S | R |  | S |
| GX219 |  |  | H | F | S | A |  |  |  |  | P | G | E | R | A | L |  | S | R |  | S |
| GX304 |  |  | H | F | S | A |  |  |  |  | P | G | E | R | A | L |  | S | R |  | S |
| GXPX |  |  | H | F | S | A |  |  |  |  | P | G | E | R | A | L |  | S | R |  | S |
| GXPXD |  |  | H | F | S | A |  |  |  |  | P | G | E | R | A | L |  | S | R |  | S |
| GXLCC |  |  | H | F | S | A |  |  |  |  | P | G | E | R | A | L |  | S | R |  | S |
| GXPL |  |  | H | F | S | A |  |  |  |  | P | G | E | R | A | L |  | S | R |  | S |
| GXYZD |  |  | H | F | S | A |  |  |  |  | P | G | E | R | A | L |  | S | R |  | S |
| GXNN2 |  | G | H | F | S | A |  |  |  |  | P | G | E | R | A | L |  | S | R |  | S |
| GXLA11 |  | G | H | F | S | A |  |  |  |  | P | G | E | R | A | L |  | S | R |  | S |
| GXBS892010 |  |  | H | F | S | A |  |  |  |  | P | G | E | R | A | L |  | S | R |  | S |
| GXBS092010 |  |  | H | F | S | A |  |  |  |  | P | G | E | R | A | L |  | S | R |  | S |
| GXLQ2010 |  |  | H | F | S | A |  |  |  |  | P | G | E | R | A | L |  | S | R |  | S |
| GXBH2011 |  |  | H | F | S | A |  |  |  |  | P | G | E | R | A | L |  | S | R |  | S |
| GXLB2010 |  |  | H | F | S | A |  |  |  |  | P | G | E | R | A | L |  | S | R |  | S |
| GXN119 | Ⅲ | D | Q | S | P | V | S | A | P |  | P | G | E | R | A | L |  | S | R | V | S |
